# Centre-Table Ornaments

**Published:** 1858-02

**Authors:** 


### CENTRE-TABLE ORNAMENTS.

CALLING not long since at the house of a literal millionaire, even after the "Panic," and, while waiting for him, we noticed a new arrangement in the parlor: a round table was in the centre, with a brilliant drop gas light from the ceiling. Of the four books on the table, we picked up one, and it proved to be a bound volume of "Hall's Journal of Health." As we had seen that very often before, and had read the whole of it, we laid it down very quickly, and took up another—it was *Hall's Journal of Health;* but wanting to see something new, we laid it down also, and took a third volume, that proved to be HALL'S JOURNAL OF HEALTH. Getting tired of the same old song, we hastily caught up the fourth, and only remaining volume, which was none other than HALL'S JOURNAL OF HEALTH. No doubt, if we had looked upon the side-table, we should have found "Hall's Bronchitis, and Kindred Diseases," and "Hall on Consumption;" but we were saved the infliction, by the entrance of one of the most courteous and warm-hearted gentlemen of our time. This incident gives us occasion to suggest what books should be most accessible to a growing up family, or to a waiting visitor. We trust that thoughtful parents will mature the idea well, and act upon it without delay; the value of it to the children, the value of it to ourselves, and the comfort to waiting visitors, in making the time appear so much shorter, by having their attention directed to some short article of practical personal interest, these things will be apparent at first glance. Let there be in the parlor, and in the most frequented family room, in useable binding, and in a kind of disorder, not stacked up in prim piles, of "gilt-edged" and "morocco," first of all "THE FAMILY BIBLE," then WEBSTER'S QUARTO DICTIONARY, unabridged; third, *Colton* or *Cowperthwaite's Folio Universal Atlas*, and *fourth*, the four bound volumes of our JOURNAL OF HEALTH, and have no other books besides these in the family-rooms just named. The reasons for the three first-mentioned volumes will suggest themselves at once. For our own, we will say, that never advising a dose of medicine, except in one single instance in four years (in case of cholera, when a physician could not be had promptly), the reading of an article would be *safe*. Besides, our articles are usually very short, and direct to the point; always on the side of good morals; advocating what is useful, temperate, methodical, honorable, and courteous; giving plain, common-sense, rational directions as to all the habits of life, eating, sleeping, exercise; the importance of guarding against taking colds, of keeping the feet warm, and of bodily regularity; and all given on white paper, and good-sized, clear type—easily read, even in a darkened parlor; these considerations make it a kind of humanity to have, at least, one of the volumes of our Journal on every centre-table in the United States; a better, and safer, and more profitable fashion than the present, of the last novel, the trashy monthly, with its really beautiful embellishments; the ghastly caricatures of the wishy-washy weeklies, with fillings-up of jokes, not only stale, but low, and very often profane: to say nothing of the inane love-stories, the "horrible revelations," "astounding developments," and column after column of details of thefts, murders, rapes, abortions, elopements, and "liaisons," with all the disgusting particularity, in which these publications delight to riot and revel! And yet, in three-fourths of the houses of New York, such publications are received every week, to be gloated over by peering girls and boys, in their teens! drank in, as the greedy fire drinks up the water, and stimulating the budding passions and propensities to a height of greed, which too often breaks through all restraint, and leaves the victims a wreck, long before they enter their majority.

